# Antifungal Therapy in *Candida* Infective Endocarditis: A Comparison of Echinocandins and Other Treatment Regimens in a Nationwide Cohort Study

**DOI:** 10.1093/cid/ciaf312

**Published:** 2025-06-14

**Authors:** Siri Kurland, Mia Furebring, Elisabeth Löwdin, Lars Olaison, Jan Sjölin

**Affiliations:** Department of Medical Sciences, Infection Medicine, Uppsala University, Uppsala, Sweden; Department of Medical Sciences, Infection Medicine, Uppsala University, Uppsala, Sweden; Department of Medical Sciences, Infection Medicine, Uppsala University, Uppsala, Sweden; Department of Infectious Diseases, Institute of Biomedicine, University of Gothenburg, Gothenburg, Sweden; Department of Medical Sciences, Infection Medicine, Uppsala University, Uppsala, Sweden

**Keywords:** *Candida*, infective endocarditis, echinocandins, amphotericin B, azoles

## Abstract

**Background:**

Amphotericin B-based treatment has been the cornerstone of *Candida* infective endocarditis (CIE) treatment, but recent guidelines also recommend echinocandins. However, there are limited outcome data. The few studies performed report differing results. Our aim in this study was to compare the outcome with echinocandins to those of other antifungal strategies.

**Methods:**

A CIE cohort was derived from the Swedish Registry of Infective Endocarditis for 1995–2019. In-hospital mortality or relapse was considered treatment failure.

**Results:**

51 episodes in 38 patients were treated with echinocandins (n = 21), amphotericin B-based therapy (n = 22), or azoles (n = 8). The proportions of treatment failure were 32%, 38%, and 62%, respectively (*P* = .35). Patients who received echinocandins were older and had a higher burden of comorbidities. The overall 1-year mortality rate from index hospitalization was 26%, with no significant differences between backbone therapies (*P* = .18). Because *Candida parapsilosis* IE was not treated with echinocandins, a subgroup analysis was performed of 33 episodes with *C.* non*-parapsilosis* that showed no significant differences between backbone therapies (*P* = .33). In a subgroup analysis of episodes treated with amphotericin B-based therapy, treatment failure was seen in 54% of 13 episodes caused by *C. parapsilosis* and 12% of 8 episodes caused by *C.* non*-parapsilosis* (*P* = .10).

**Conclusions:**

No differences in in-hospital mortality, relapse rate, or 1-year mortality were seen in patients with echinocandin-based therapy compared with other CIE regimens, despite risk factors in the echinocandin group that would be expected to negatively bias outcome. *Candida parapsilosis* CIE appears to pose specific challenges in terms of optimal therapy.

## Teaser

The choice of echinocandin or amphotericin B-based therapy did not impact the outcome in in-hospital mortality or relapse rate in patients with *Candida* infective endocarditis (CIE). *Candida parapsilosis* CIE appears to pose specific challenges in terms of optimal therapy.

## Graphical Abstract

**Graphical Abstract:**
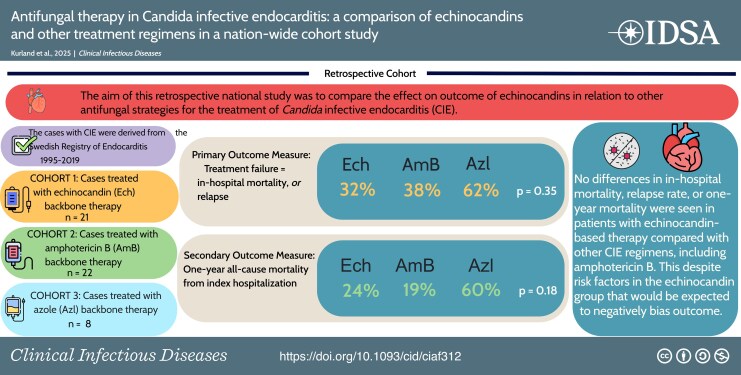
https://tidbitapp.io/institutional-portal/clinical-infectious-diseases/tidbits/antifungal-therapy-in-candida-infective-endocarditis-a-comparison-of-echinocandins-and-other-treatment-regimens-in-a-nation-wide-cohort-study-04f4133d-d341-435c-8c21-fe31e46ed5db/update

*Candida* infective endocarditis (CIE) is a rare presentation of invasive candidiasis that is associated with high mortality rates of 30%–57% [1–5]. Although CIE accounts for less than 2% of all cases of IE, the at-risk population is increasing as a result of advances in medical therapies and interventions [6]. Many *Candida* species have excellent biofilm-forming capacity, and the vegetations in CIE exhibit properties that resemble those of biofilms, in particular, a recalcitrant nature to antimicrobial therapy [7–10].

When treating patients with CIE, the clinician is faced with a range of challenging situations, such as high burden of comorbidity, unsatisfactory treatment response, and potential side effects, that often lead to a need for multiple alterations in antifungal therapy and long periods of hospitalization. The standard of care is antifungal therapy in combination with surgery [11–14]. The armamentarium of antifungals is limited, but echinocandins have been used increasingly in recent years. Current guidelines stipulate lipid formulations of amphotericin B with or without flucytosine or echinocandins as first-line therapy [12, 14]. It has not been elucidated if one of these antifungals is superior to the other or if there are specific patient- or species-related factors to be taken into consideration in the choice of antifungal. The rarity of CIE limits the possibility of conducting randomized, controlled studies to compare antifungals' impact on mortality and relapse. Therefore, recommendations are, to a large extent, based on retrospective case series and expert opinions. In recent publications of observational data, comparisons between amphotericin B-based and echinocandin-based therapies have been performed with somewhat diverging results [3, 5, 15]. In summary, no data that compare the impact on outcome have unequivocally demonstrated the superiority of either antifungal. Thus, as of now, the optimal management and choice of antifungal strategy in CIE remains to be clarified.

Our aim in this national study was to compare the effect on outcome of echinocandins in relation to other antifungal strategies for the treatment of CIE.

## METHODS

### Study Population and Data Collection

We conducted a national study on patients with CIE diagnosed between January 1995 and December 2019. Cases were derived from the Swedish Registry of Infective Endocarditis (SRIE), which holds data from patients treated for IE in Sweden since 1995. The departments of infectious diseases in Sweden report consecutive cases through a standardized case report form that records more than 200 variables on patient characteristics, management, and outcome. Additionally, the patients' medical records were retrospectively reviewed. For information on date and cause of death, the National Cause of Death Register, managed by the National Board of Health and Welfare, was accessed in January 2022.

Data collected included demographics, comorbidities applied in the Charlson comorbidity index (CCI), presence and type of prosthetic valve and/or cardiac implantable electronic device (CIED), and microbiological data. Predisposing conditions were registered, including antibiotic treatment, central venous catheter, parenteral nutrition, admission to an intensive care unit, abdominal surgery, use of corticosteroids, and cancer treatment, as well as neutropenia, all occurring within 3 months prior to the onset of signs or symptoms consistent with CIE. Clinical data covered signs and symptoms, diagnostics, clinical complications, antifungal therapy, surgery, and outcome.

### Definitions and Categorizations

CIE was classified as definite or possible IE based on the modified Duke criteria [16]. Microbiological evidence of *Candida* spp. in blood or valve cultures was required. The infection was defined as either community-associated or healthcare-associated, as previously proposed [17]. The antifungal drug that the patient received for the majority of the first 30 days of treatment was considered the backbone therapy [3]. Based on this, patients were assigned to the echinocandin-based, amphotericin B-based or azole-based backbone treatment group. A backbone combination regimen was defined as 2 antifungals given concomitantly for the majority of the first 30 days of treatment. Any combination therapy was defined as receiving 2 antifungals concomitantly for more than 1 day, irrespective of time point. Amphotericin B-based therapy included amphotericin B deoxycholate or liposomal amphotericin B. According to European guidelines, during the study period, echinocandins were not considered a treatment option for severe invasive *Candida* infections with *Candida parapsilosis* because of the high minimum inhibitory concentrations [18].

Suppressive therapy was any antifungal intended to prevent relapses, with a required minimum duration of use of 3 months. In cases where azoles or echinocandins were prescribed for both treatment and suppression, the transition was considered to occur at discharge, with a required duration of more than 3 months post-discharge.

The primary outcome was treatment failure, defined as in-hospital mortality or relapse. The secondary outcome was 1-year all-cause mortality from the date of admission for the index hospitalization. An episode was defined as an admission for CIE, regardless of whether it was the patient's index CIE or a relapse. A relapse was a new episode of CIE due to the same *Candida* spp. obtained in blood cultures in a patient with clinical recovery during index hospitalization and subsequent discharge. No molecular method was used to confirm whether the *Candida* strains were identical or not. The primary analysis was performed on all episodes. Because echinocandins were not used in *C. parapsilosis* IE, a secondary subgroup analysis for treatment failure was carried out on episodes caused by *C.* non*-parapsilosis* spp. We applied a 2-year follow-up period for each episode.

The Uppsala University's local ethics committee and the Swedish Ethical Review Authority approved the study.

### Statistical Analyses

In the outcome analysis, a binary logistic mixed-effects regression model (generalized linear mixed model) was used to analyze the episodes, taking into account that each individual could relapse and therefore occur in multiple episodes. In the model, the dependent variables were relapse, in-hospital mortality, or treatment failure. The fixed effect was the backbone therapy, and the random effect was the individual.

Continuous data are presented as medians and interquartile ranges (IQRs), unless otherwise indicated, and compared using the Mann–Whitney *U* test and Kruskal–Wallis analysis of variance. Categorical data are presented as numbers and percentages and compared using the *χ*^2^ test and Fisher exact test, as appropriate. A *P* value of <.05 was considered significant. Data were analyzed using Data Science Workbench (version 14, TIBCO Software Inc, 2020) and SPSS software (version 28, IBM, Armonk, NY).

## RESULTS

A total of 39 patients with CIE were reported. One patient died prior to initiation of antifungals and was therefore excluded. This resulted in 38 patients: 36 were classified as having definitive CIE and 2 as possible CIE. Nine patients (24%) experienced a total of 13 relapses, resulting in 51 episodes of CIE in total.

Baseline characteristics of the 38 patients are summarized in Table 1, grouped by backbone therapy. The corresponding data for the 51 episodes are presented in Supplementary Table 1. Backbone regimens were distributed as follows: echinocandins in 22 episodes, amphotericin B-based therapy in 21 episodes, and azoles in 8 episodes. There were no significant differences between the groups, but the patients who received echinocandins were older and had a higher median CCI.

**Table 1.** Baseline Characteristics at Index Hospitalization in the Overall Cohort of 38 Patients With *Candida* Infective Endocarditis and Comparison by Backbone Therapy

Baseline Characteristic	Overall Cohort N = 38	Echinocandin n = 17	Amphotericin B n = 16	Azole n = 5	*P* Value^a^
Age, (IQR), y	62 (48–70)	66 (58–74)	56 (47–62)	65 (46–69)	.14
Sex, male/female	32/6	15/2	13/3	4/1	.83
Healthcare-associated IE	28 (74)	14 (82)	9 (56)	5 (100)	.08
Comorbidities and other predisposing factors					
Charlson comorbidity index, median (IQR)	4 (1–7)	6 (5–7)	1.5 (0–4)	3 (1–6)	.05
Chronic renal failure	11 (29)	8 (47)	3 (19)	…	.06
Chronic heart failure	9 (24)	6 (35)	3 (19)	…	.22
Chronic obstructive pulmonary disease	2 (5)	1 (6)	…	1 (20)	.21
Diabetes mellitus	8 (21)	6 (35)	2 (12)	…	.13
Liver, chronic disease	7 (18)	2 (12)	3 (19)	2 (40)	.36
Prosthetic valve^b^	20 (53)	8 (47)	10 (62)	2 (40)	.56
CIED	5 (13)	4 (24)	2 (12)	…	.40
Previous bacterial IE	16 (42)	7 (41)	6 (38)	3 (60)	.67
Congenital heart disease	2 (5)	…	2 (12)	…	.23
Persons who inject drugs	14 (37)	5 (29)	6 (38)	3 (60)	.46
Central venous line <3 mo prior to CIE	20 (53)	10 (59)	6 (38)	4 (80)	.20
Antibiotics <3 mo prior to CIE	25 (66)	13 (76)	9 (56)	3 (60)	.45
Intensive care unit <3 mo prior to CIE	9 (24)	7 (41)	2 (12)	…	.06
Cancer, solid or hematological^c^	8 (21)	4 (24)	3 (19)	1 (20)	.94
Neutropenia <3 mo prior to CIE	1 (3)	1 (6)	…	…	.53
Corticosteroids <3 mo prior to CIE	6 (16)	2 (12)	3 (19)	1 (20)	.83
Year of hospitalization, median (IQR)	2013 (2010–2016)	2014 (2012–2016)	2010 (1997–2017)	2012 (2002–2013)	.19
Type of IE^d^					
Native valve endocarditis left-sided	11 (29)	4 (24)	5 (31)	2 (40)	.75
Native valve endocarditis right-sided	7 (18)	3 (18)	3 (19)	1 (20)	.99
Prosthetic valve endocarditis left-sided	17 (45)	7 (41)	8 (50)	2 (40)	.86
CIED-IE	5 (13)	4 (24)	1 (6)	…	.22
*Candida* species					
*Candida albicans*	20 (53)	13 (76)	4 (25)	3 (60)	.01
*Candida parapsilosis*	10 (26)	…	8 (50)	2 (40)	Not analyzed
*Nakaseomyces glabrata*	6 (16)	4 (24)	2 (12)	…	.40
*Candida tropicalis*	1 (3)	…	1 (6)	…	.50
*Candida pelliculosa*	1 (3)	…	1 (6)	…	.50

Data are presented as no. (%), unless otherwise specified.

Abbreviations: CIE, *Candida* infective endocarditis; CIED, cardiac implantable electronic device; IE, infective endocarditis; IQR, interquartile range.

^a^ *P* values for comparison between the 3 groups: echinocandin, amphotericin B, and azole.

^b^Three patients had a prosthetic valve, but the CIE affected a native valve.

^c^Hematological cancer—1 patient with myeloma, no bone marrow recipient.

^d^Two patients had a combination of CIED-IE and valvular endocarditis.

### Mycology

*Candida albicans* was the most common species isolated from 20 patients (53%), followed by *C. parapsilosis* (n = 10, 26%) and *Nakaseomyces glabrata* (*Candida glabrata*) (n = 6, 16%). *Candida tropicalis* and *Candida pelliculosa* were isolated in 1 patient each (Table 1). Blood cultures were positive in 48 episodes. In the remaining 3, *Candida* spp. were identified from excised cardiac valves. In 20 of the 21 episodes that underwent valve surgery, the cardiac tissue yielded positive cultures. Fifteen of these episodes had received antifungals prior to surgery, for a median duration of 16 days (IQR, 9–24). All *Candida* spp. were susceptible to the backbone treatment prescribed in each given episode.

### Management

Overall, 24 (47%) episodes were managed with antifungal therapy alone, 21 episodes (41%) underwent adjunctive valve surgery, and all 6 (12%) with CIED-IE underwent device extraction. No statistically significant differences in management strategy were observed with respect to the backbone therapy groups (Table 2). The corresponding data based on the 38 patients' index hospitalizations are presented in Supplementary Table 2.

**Table 2.** Clinical Complications and Management in 51 Episodes of *Candida* Infective Endocarditis and Comparison by Backbone Therapy

Complications and management	Overall Cohort N = 51	Echinocandin n = 22	Amphotericin B n = 21	Azole n = 8	*P V*alue^a^
Intracardiac complications					
Regurgitation	24 (47)	9 (41)	9 (43)	6 (75)	.22
Perivalvular abscess	9 (18)	4 (18)	4 (19)	1 (12)	.91
Paravalvular leakage prosthetic valve	5 (10)	3 (14)	2 (10)	…	.54
Clinical complications					
Heart failure	17 (33)	8 (36)	6 (29)	3 (38)	.38
Kidney failure^b^	18 (35)	5 (23)	11 (55)	2 (33)	.10
Embolization	29 (57)	7 (32)	15 (71)	7 (88)	.0052
Central nervous system	9 (18)	…	8 (38)	1 (12)	.0053
Eye manifestation^c^	5/38	1/16	4/16	−/6	.17
Management					
Antifungal therapy only	24 (47)	8 (36)	10 (48)	6 (75)	.17
Adjunctive valve surgery	21 (41)	9 (41)	10 (48)	2 (25)	.54
Cardiac implantable electronic device extraction	6 (12)	5 (23)	1 (5)	…	.10
Duration of antifungal treatment,^d^ d	56 (42–67)	58 (45–68)	49 (42–66)	54 (43–62)	.54
Antifungal backbone combination	6 (12)	…	6 (29)	…	.01
Any antifungal combination	13 (25)	…	12 (57)	1 (12)	.00
Suppressive therapy^e^	31 (70)	13 (68)	15 (79)	3 (50)	.39

Data are presented as no. (%) or median (interquartile range).

^a^ *P* values for comparison between the 3 groups: echinocandin, amphotericin B, and azole.

^b^Missing data for 3 episodes.

^c^Based on ophthalmoscopy performed in 38 of the episodes.

^d^Antifungal treatment defined as treatment aimed for cure, that is, during hospitalization and outpatient therapy, excluding suppressive therapy.

^e^The % is based on the 44 episodes alive at discharge and thereby eligible for suppressive therapy.

The median time from blood culture to initiation of antifungal therapy was 3 days (IQR, 2–5), and time from blood culture to start of backbone therapy was 5 days (IQR, 3–7.5). The most frequently used initial antifungal was an echinocandin (n = 28), followed by an azole (n = 12). Six of the 12 episodes initially treated with azoles were switched to echinocandin or amphotericin B-based backbone therapy after a median of 7 days (IQR, 4–8.5). A majority, 28 episodes, received multiple antifungal regimens with varying duration during hospitalization.

Amphotericin B-based therapy was used at some point during the course of treatment in 25 of the episodes. In 11 of them, therapy discontinuation or dose reduction was performed due to renal deterioration. Infusion-related side effects resulted in the discontinuation of amphotericin B-based therapy in 1 episode. Termination of therapy due to adverse reactions was not observed in any of the episodes treated with echinocandins. Six episodes (12%) treated with amphotericin B were put on additional concomitant antifungal therapy: flucytosine in 4 episodes and fluconazole in 2.

At discharge, 31 of 44 episodes obtained suppressive therapy (azoles, n = 29; echinocandins, n = 2; Table 2).

### Outcome

Treatment failure frequency in episodes with echinocandin-based, amphotericin B-based, and azole-based backbone therapy did not differ significantly, being 32%, 38%, and 62%, respectively (*P* = .35; Table 3). Sensitivity analysis that separated the 2 amphotericin B formulations yielded the same result. Also, there were no significant differences between backbone groups regarding the individual components of in-hospital mortality (14%, 10%, and 25%, respectively; *P* = .56) or relapse (18%, 29%, and 38%, respectively; *P* = .46). In addition, outcome analysis that included all individual treatment regimens of varying duration did not result in significant differences (*P* = .30). In Supplementary Table 3, outcomes in the 38 patients' index episodes and any subsequent relapses are shown separately. The 1-year mortality rate from index hospitalization was 26%. The rates did not differ significantly between backbone therapies (*P* = .18; Table 3).

**Table 3.** Outcome by Backbone Treatment in 51 Episodes of *Candida* Infective Endocarditis

Outcome	Overall Cohort N = 51	Echinocandin n = 22	Amphotericin B n = 21	Azole n = 8	*P* Value^a^
Treatment failure^b^	20 (39)	7 (32)	8 (38)	5 (62)	.35
In-hospital mortality	7 (14)	3 (14)	2 (10)	2 (25)	.56
Relapse	13 (25)	4 (18)	6 (29)	3 (38)	.46^c^
One-year mortality from index hospitalization^d^	10 (26)	4 (24)	3 (19)	3 (60)	.18

Data are represented as no. (%).

^a^ *P* values for comparison between the 3 groups: echinocandin, amphotericin B, and azole.

^b^Treatment failure defined as in-hospital mortality or relapse.

^c^ *P* value based on 44 episodes alive at discharge.

^d^The % is based on the 38 patients in the overall cohort.

In the secondary analysis of the 33 episodes of *C.* non-*parapsilosis* IE, treatment failure did not differ significantly with regard to backbone therapy (*P* = .33; Table 4). Baseline characteristics of particular interest are shown in Supplementary Table 4. Among the 21 episodes who received amphotericin B-based backbone therapy, a larger proportion with *C. parapsilosis* IE than with *C.* non*-parapsilosis* IE suffered treatment failure: 7 of 13 and 1 of 8, respectively, without major differences in confounding factors (Supplementary Table 5). However, statistical significance was not reached (*P* = .10).

**Table 4.** Outcome by Backbone Treatment in 33 Episodes of *Candida* Non*-Parapsilosis* Infective Endocarditis Subgroup Analysis

Outcome	Overall Cohort N = 33	Echinocandin n = 22	Amphotericin B n = 8	Azole n = 3	*P* Value^a^
Treatment failure^b^	10 (30)	7 (32)	1 (12)	2 (67)	.33
In-hospital mortality	5 (15)	3 (14)	…	2 (67)	.02
Relapse	5 (15)	4 (18)	1 (12)	…	.91^c^
One-year mortality from index hospitalization^d^	7 (25)	4 (24)	1 (12)	2 (67)	.17

Data are presented as no. (%).

^a^ *P* values for comparison between the 3 groups: echinocandin, amphotericin B, and azole.

^b^Treatment failure defined as in-hospital mortality or relapse.

^c^ *P* value based on 28 episodes alive at discharge.

^d^The % is based on the 28 patients with *Candida* non*-parapsilosis* infective endocarditis.

Among the 12 episodes in the overall cohort who received an azole as first-line therapy, 6 (50%) experienced treatment failure compared with 14 of 39 (36%) who received an echinocandin or amphotericin B-based drug as first-line therapy (*P* = .40).

## DISCUSSION

Studies comparing antifungals in CIE are few and of limited size. In this registry-based study of 38 patients with 51 CIE episodes, where 22 episodes were treated with an echinocandin as backbone therapy, we report the outcome with a focus on antifungal therapy. In previous reports, Arnold et al studied 33 patients and Rivoisy et al studied 46 patients, of whom 9 and 10, respectively, received an echinocandin as single treatment [3, 5]. In the present study, the choice of echinocandin-based or amphotericin B-based backbone therapy did not significantly impact in-hospital mortality or relapse rate. These results are despite the fact that patients treated with an echinocandin-based regimen were older and had a higher burden of comorbidity, which would be expected to skew the outcome to the disadvantage of echinocandins. Additionally, healthcare-associated IE, which has been reported to have a higher mortality rate [2], was more common in the patients treated with an echinocandin. Because none of the patients with *C. parapsilosis* IE episodes received an echinocandin, further exploration of the data was performed in patients with *C.* non*-parapsilosis* IE, aiming to compare backbone therapies without the risk of species bias. No differences in outcome that achieved significance were observed in that analysis. Our results are in line with those of Arnold et al, in which a subgroup analysis did not show any difference in mortality between echinocandin-based and amphotericin B-based therapies [3]. In that study, as in our study, the patients on echinocandin-based therapy had factors expected to bias toward a poorer outcome. These results are contrasted by those of Rivoisy et al, who reported a significantly improved 6-month survival rate in 46 patients with prosthetic valve CIE when treated with liposomal amphotericin B-based regimens compared with echinocandins or other antifungals [5]. These diverging results can be attributed to a number of factors including limited sample sizes, dissimilar proportions of the type of CIE, and varying distributions of *Candida* spp. with different abilities for biofilm formation.

Whether or not IE is a biofilm infection is debatable, but it undoubtedly has features consistent with biofilm infection [7]. This is underlined by the superior effect of biofilm-active antifungal therapies compared with azoles in the management of CIE [15]. Our results with poorer outcome in azole-treated patients are in line with this, although the number of patients was too small to reach significance. Echinocandins have activity against *Candida* biofilm in vitro [10, 19, 20], and their role in the treatment of invasive *Candida* infections is further supported by their safety profile [21, 22]. Although conventional amphotericin B has inferior biofilm activity in vitro compared with echinocandins, lipid amphotericin B formulations exhibit superior biofilm activity compared with that of conventional amphotericin B [19]. In all, the results reported in the present and aforementioned studies could potentially indicate that there is no major difference in efficacy between echinocandin-based and amphotericin B-based regimens for the treatment of *C.* non*-parapsilosis*, provided that liposomal amphotericin B is used. The differing results could then mainly be attributed to the patient compositions in the relatively small samples.

Regarding *C. parapsilosis*, none of the CIE patients in the present study received an echinocandin as backbone regime, in accordance with the guidelines in Europe at the time. In recent international guidelines, there is less reluctance to use echinocandins for the treatment of *C. parapsilosis* [12, 18]. However, the European Committee on Antimicrobial Susceptibility Testing still urges caution with echinocandins for serious infections caused by *C. parapsilosis* [18]. CIE is a serious complication of candidemia, and the proposed prudence is supported by the slower mycological clearance of *C. parapsilosis* seen in candidemia studies [23]. In the study by Rivoisy et al in which *C. parapsilosis* was the most common causative species (41%), the response to echinocandins was inferior to the response to liposomal amphotericin B, with a 6-month mortality that reached 37% [5]. There was no detailed information concerning which species were treated with echinocandins, but it could be speculated that *C. parapsilosis*' poor response to echinocandins might have contributed to this result. Regarding the effect of amphotericin B-based therapy on *C. parapsilosis*, in the present study, we cannot exclude that this antifungal might also have a poorer response. However, in contrast to what is the case for echinocandins, this was not observed in the candidemia studies [22, 24, 25]. Additionally, in animal experiments, amphotericin B has been found to exert a stronger fungicidal effect in *C. parapsilosis* than echinocandins at concentrations that correspond to those used in patients [26].

Giuliano et al showed that echinocandin or amphotericin B-based therapy reduced the likelihood of death, whereas azoles in monotherapy did not [15]. Thus, it may be speculated that initial treatment with azoles might exert a detrimental effect irrespective of subsequent treatment. However, in the present study, we did not find evidence for this, provided azole treatment duration was confined to 1 week or less.

Interestingly, the large majority of valve cultures displayed growth despite preoperative antifungal treatment. Positive valve cultures in bacterial IE have been identified as a risk factor for negative outcome [27, 28]. Whether or not positive valve cultures in CIE impact survival could not be addressed in the present study. However, the fact that preoperative antifungal treatment was administered with a median duration of 16 days, with 1 patient displaying positive valve cultures despite 52 days of preoperative therapy, emphasizes the recalcitrant nature of CIE. This condition requires highly effective and extended antifungal treatment, as well as suppressive therapy.

One-year mortality in the present study was lower than previously reported, making differences more difficult to detect. Arnold et al reported in-hospital mortality and 1-year mortality of 36% and 59%, respectively [3]. A factor that potentially impacted the mortality rate in the present study was the large proportion of persons who inject drugs (PWID), constituting 37% compared with 11% in the study by Arnold et al. The PWID in the present study were younger than the non-PWID, and none of them died during hospitalization (data not presented). Our results are in line with those of Lefort et al, who also found that PWID had better outcomes than non-PWID [2].

This study has the limitations of any observational study with a small sample size. First, the small number of patients with CIE precludes any robust statistical conclusions. Further, although all of the departments of infectious diseases in Sweden have committed to reporting cases to the SRIE, doing so is optional, and reporting bias cannot be ruled out. However, this national study reports on patients from all infectious diseases departments, not only referral centers. Last, data were reported to the registry by different physicians, which could affect data quality. This issue was at least partially compensated for through our exhaustive analysis of medical records and gathering date and cause of death from the National Cause of Death Register. Given the current lack of randomized studies, detailed observational studies comprise a plausible basis for drawing tentative conclusions.

## CONCLUSIONS

Our data add to those previously published. No differences in treatment failure or 1-year mortality were found between patients with CIE who received echinocandin-based therapy and those on amphotericin B-based therapy, despite the risk factors of the former group, which would be expected to negatively bias outcome. *Candida parapsilosis* IE poses specific challenges in terms of identifying the optimal therapy. Collaborative approaches are needed in future studies to validate and expand optimal strategies in the management of CIE.

## Supplementary Material

ciaf312_Supplementary_Data

## Supplementary Data

Supplementary materials are available at *Clinical Infectious Diseases* online. Consisting of data provided by the authors to benefit the reader, the posted materials are not copyedited and are the sole responsibility of the authors, so questions or comments should be addressed to the corresponding author.

## Notes

***Acknowledgmen*** ***ts***. The authors thank the following colleagues for their invaluable assistance in providing supplementary clinical information: Drs Björn Diedrichs, Anita Hellgren, Jan Källman, Joakim Lundvik, Maria Palmérus, Magnus Rasmussen, and Martin Widlund.

***Data availability*.** Anonymous data will be provided by the corresponding author upon reasonable request.

***Financial support.*** This work was supported by the Uppsala University Hospital Research Fund.
