# Supplementary material for: Antifungal Therapy in Candida Infective Endocarditis: A Comparison of Echinocandins and Other Treatment Regimens in a Nationwide Cohort Study
Source: Clin Infect Dis. 2025 Jun 14;82(3):e455–61. doi: 10.1093/cid/ciaf312 (PMC13017458; doi:10.1093/cid/ciaf312)
Supplement: ciaf312_Supplementary_Data [file ciaf312_supplementary_data.docx]

**Supplemental Material**

**Manuscript Title:
Antifungal therapy in *Candida* infective endocarditis: a comparison of echinocandnins and other treatment regimens in a nation-wide cohort study**

**List of Supplemental Tables**

**Table S1.** Baseline characteristics of 51 episodes in 38 patients at index hospitalization
 with *Candida* infective endocarditis and comparison by backbone therapy.

**Table S2.** Clinical complications and management at index hospitalization in the overall
 cohort of 38 patients with Candida infective endocarditis and comparison by
 backbone therapy.

**Table S3.** Treatment failure in 51 episodes and one-year mortality in 38 patients with
 *Candida* infective endocarditis.

**Table S4.** Characteristics of particular interest in 33 episodes of *Candida non*
 *parapsilosis* endocarditis and comparison by backbone therapy.

**Table S5.** Characteristics of particular interest and outcome in 21 episodes of *Candida*
 infective endocarditis receiving amphotericin B-based backbone therapy, with
 comparison between *C. parapsilosis* and *C. non-parapsilosis.*

| **Table S1. Baseline characteristics of 51 episodes in 38 patients at index hospitalization with *Candida* infective endocarditis and comparison by backbone therapy.** | | | | |
| --- | --- | --- | --- | --- |
|  | **Echinocandin**  n=22 | **Amphotericin B**  n=21 | **Azole**  n=8 | *p-*value^a^ |
| **Baseline characteristics** |  |  |  |  |
| Age, years, median (IQR) | 66 (50–74) | 56 (48–58) | 50 (46–67) | 0.11 |
| Weight, kg, median (IQR) | 80 (71–85) | 75 (57–80) | 68 (66–83) | 0.18 |
| Sex (male/female) | 19/3 | 16/5 | 7/1 | 0.63 |
| Healthcare-associated IE | 18 (82) | 10 (48) | 6 (75) | 0.05 |
| CCI, median (IQR) | 6 (1–7) | 2 (0–3) | 1.5 (0.5–4.5) | 0.05 |
| Chronic renal failure | 10 (45) | 3 (14) | 1 (12) | 0.04 |
| Chronic heart failure | 8 (36) | 5 (24) | 2 (25) | 0.64 |
| COPD | 1 (5) | - | 1 (12) | 0.29 |
| Diabetes mellitus | 7 (32) | 3 (14) | - | 0.11 |
| Liver, chronic disease | 2 (9) | 3 (14) | 2 (25) | 0.53 |
| **Predisposing conditions** |  |  |  |  |
| Prosthetic valve^b^ | 12 (55) | 13 (62) | 5 (62) | 0.86 |
| CIED | 5 (23) | 1 (5) | - | 0.10 |
| Previous bacterial IE | 9 (41) | 7 (33) | 5 (83) | 0.09 |
| Congenital heart disease | - | 3 (14) | 2 (25) |  |
| Persons who inject drugs | 7 (32) | 9 (43) | 6 (75) | 0.11 |
| Central venous line <3 mo prior to CIE | 10 (45) | 8 (38) | 5 (62) | 0.50 |
| Antibiotics <3 mo prior to CIE | 14 (64) | 9 (43) | 4 (50) | 0.39 |
| ICU <3 mo prior to CIE | 7 (32) | 2 (10) | - | 0.06 |
| Cancer, solid or hematological | 4 (18) | 4 (19) | 1 (12) | 0.91 |
| Neutropenia <3 mo prior to CIE | 1 (4) |  |  | 0.51 |
| Corticosteroids <3 mo prior to CIE | 4 (18) | 4 (19) | 1 (12) | 0.91 |
| **Year of hospitalization,**  median (IQR) | 2015  (2012–2017) | 2009 (1998–2018) | 2010  (2007–2014) | 0.45 |
| **Type of infection**^d^ |  |  |  |  |
| NVE left-sided | 4 (18) | 6 (29) | 2 (25) | 0.72 |
| NVE right-sided | 4 (18) | 4 (19) | 1 (12) | 0.91 |
| PVE left-sided | 10 (45) | 11 (52) | 5 (62) | 0.70 |
| CIED-IE | 5 (23) | 1 (5) | - | 0.1 |
| ***Candida* species** | - | - | - |  |
| *Candida albicans* | 17 (77) | 4 (19) | 3 (38) | <0.001 |
| *Candida parapsilosis* | - | 13 (62) | 5 (62) | NA |
| *Nakaseomyces glabrata* | 5 (23) | 2 (9) | - | 0.21 |
| *Candida tropicalis* | - | 1 (5) | - | 0.48 |
| *Candida pelliculosa* | - | 1 (5) | - | 0.48 |
| Data are presented as no (%), unless otherwise specified.  CCI, Charlson Comorbidity Index; CIE, candida infective endocarditis; CIED, cardiac implantable electronic device; CIED-IE, cardiac implantable electronic device infective endocarditis; COPD, chronic obstructive pulmonary disease; d, days; ICU, intensive care unit; IE, infective endocarditis; IQR, interquartile range;  kg, kilogram; mo, months; NA, not analyzed; NVE, native valve endocarditis; PVE, prosthetic valve endocarditis. ^a^*p*-values for comparison between the three groups amphotericin B, echinocandin and azole. ^b^Four patients had a prosthetic valve, but the CIE affected a native valve or a CIED.  ^c^ Hematological cancer - one patient with myeloma, no bone marrow recipient. ^d^Two patients had a combination of CIED-IE and valvular endocarditis. | | | | |

| **Table S2.** **Clinical complications and management at index hospitalization in the overall cohort of 38 patients with *Candida* infective endocarditis and comparison by backbone therapy.** | | | | | |
| --- | --- | --- | --- | --- | --- |
|  | **Overall cohort**  n=38 | **Echinocandin**  n=17 | **Amphotericin B**  n=16 | **Azole**  n=5 | *p*-value^a^ |
| **Intracardiac complications** |  |  |  |  |  |
| Regurgitation | 21 (55) | 8 (47) | 8 (50) | 5 (100) | 0.1 |
| Perivalvular abscess | 8 (21) | 3 (18) | 4 (25) | 1 (20) | 0.87 |
| Paravalvular leakage PVE | 4 (11) | 2 (12) | 2 (12) | - | 0.71 |
| **Clinical complications** |  |  |  |  |  |
| Heart failure | 12 (32) | 7 (41) | 4 (25) | 1 (20) | 0.51 |
| Kidney failure | 15 (39) | 5 (29) | 9 (56) | 1 (20) | 0.18 |
| Embolization | 24 (63) | 6 (35) | 14 (88) | 4 (80) | 0.006 |
| Central nervous system | 9 (24) | - | 8 (50) | 1 (20) | 0.003 |
| Eye manifestation^b^ | 5/31 | 1/14 | 4/13 | -/4 | 0.16 |
| **Management** |  |  |  |  |  |
| Antifungal therapy only | 17 (45) | 7 (41) | 6 (38) | 4 (80) | 0.23 |
| Adjunctive valve surgery | 16 (42) | 6 (35) | 10 (56) | 1 (20) | 0.27 |
| CIED extraction | 5 (13) | 4 (24) | 1 (6) | - | 0.22 |
| Duration of antifungal treatment^c^, d | 56 (42–65) | 57 (46–63) | 52 (42–66) | 44 (42–57) | 0.71 |
| Antifungal backbone combination | 5 (13) | - | 5 (31) | - | 0.02 |
| Any antifungal combination | 10 (26) | - | 9 (56) | 1 (20) | 0.001 |
| Suppressive therapy^d^ | 24 (72) | 11 (73) | 11 (73) | 2 (67) | 0.97 |
| Data are presented as no (%) or median (interquartile range). CIED, cardiac implantable electronic device; d, days; PVE, prosthetic valve endocarditis. ^a^*p*-values for comparison between the three groups amphotericin B, echinocandin and azole. ^b^Based on ophthalmoscopy performed in 31 of the episodes.  ^c^Antifungal treatment defined as treatment aimed for cure i.e. during hospitalization and out-patient therapy, excluding suppressive therapy. ^d^(%) based on the 33 patients alive at discharge and thereby eligible for suppressive therapy. | | | | | |

**Table S3.** **Treatment failure in 51 episodes and one-year mortality in 38 patients with *Candida* infective endocarditis.**

|  | | **Backbone treatment** | | | | | | | | | |
| --- | --- | --- | --- | --- | --- | --- | --- | --- | --- | --- | --- |
|  |  | **Echinocandin**  n=17 | | | **Amphotericin B group**  n=16 | | | **Azole**  n=5 | | | |
| Treatment failure | 14 (37) | 5 (29) | | | 6 (38) | | | 3 (60) | | |  |
| In-hospital  mortality | 5 | 2 | | | 1 | | | 2 | | |  |
| Relapse 1 | 9 | 3 | | | 5 | | | 1 | | |  |
| Relapse 1  Backbone  treatment |  | Amph B  n=0 | Echinocandin  n=3 | Azole  n=0 | Amph B  n=3 | Echinocandin  n=1 | Azole  n=1 | Amph B  n=0 | Echinocandin  n=0 | Azole  n=1 |  |
| Treatment failure | 4 | - | 1 | - | 2 | 0 | 1 |  |  | 0 |  |
| In-hospital  mortality | 1 |  |  |  | 1 |  |  |  |  |  |  |
| Relapse 2 | 3 |  | 1 |  | 1 |  | 1 |  |  |  |  |
| Relapse 2  Backbone  treatment |  | Amph B  n=0 | Echinocandin  n=1 | Azole  n=0 | Amph B  n=1 | Echinocandin  n=0 | Azole  n=1 | Amph B  n=0 | Echinocandin  n=0 | Azole  n=0 |  |
| Treatment failure | 2 |  | 1 |  | 0 |  | 1 |  |  |  |  |
| In-hospital  mortality | 1 |  | 1 |  |  |  |  |  |  |  |  |
| Relapse 3 | 1 |  |  |  |  |  | 1 |  |  |  |  |
| Relapse 3  Backbone treatment  and outcome |  |  | | | Relapse 3 was successfully treated with amphotericin B | | |  | | |  |
| One-year all-cause mortality^1^ | 10 (26) | 4 (24) | | | 3 (19) | | | 3 (60) | | |  |

Values are presented as no (%). ^1^One-year mortality (all-cause) applies to each patient’s index hospitalization for *Candida* infective endocarditis.

| **Table S4. Characteristics of particular interest in 33 episodes of *Candida non*-*parapsilosis* endocarditis and comparison by backbone therapy** | | | | | |
| --- | --- | --- | --- | --- | --- |
|  | **Overall cohort**  n=33 | **Echinocandin**  n=22 | **Amphotericin B**  n=8 | **Azole**  n=3 | *p*-value^a^ |
| Age, years, median (IQR) | 65 (46–74) | 66 (50–74) | 52 (43–66) | 69 (46–76) | 0.45 |
| Healthcare-associated IE | 27 (81) | 18 (82) | 6 (75) | 3 (100) | 0.63 |
| CCI, median (IQR) | 6 (1–7) | 6 (1–7) | 1 (0–6) | 6 (1–9) | 0.21 |
| PVE | 18 (54) | 10 (45) | 6 (75) | 2 (67) | 0.32 |
| Surgery | 8 (24) | 9 (41) | 4 (50) | - | 0.31 |
| Suppressive therapy^b^ | 21 (75) | 13 (68) | 7 (88) | 1 (100) | 0.49 |
| Data are presented as no (%), unless otherwise specified. CCI, Charlson Comorbidity Index; IE, infective endocarditis; IQR, interquartile range; PVE, prosthetic valve endocarditis. ^a^*p*-values for comparison between the three groups amphotericin B, echinocandin and azole. ^b^(%) based on 28 episodes who were alive at discharge and thereby eligible for suppressive therapy. | | | | | |

| **Table S5. Characteristics of particular interest and outcome in 21 episodes of *Candida* infective endocarditis receiving amphotericin B-based backbone therapy, with comparison between *C. parapsilosis* and *C. non-parapsilosis.*** | | | | |
| --- | --- | --- | --- | --- |
|  | **Overall cohort**  **amphotericin B**  **backbone**  n=21 | ***C. parapsilosis***  n=13 | ***C. non-parapsilosis***  n=8 | *p*-value^a^ |
| Age, years, median (IQR) | 56 (48–58) | 56 (50–57) | 52 (43–66) | 0.65 |
| Healthcare-associated IE | 10 (48) | 4 (31) | 6 (75) | 0.08 |
| CCI, median (IQR) | 2 (0–3) | 2 (1–3) | 1 (0–5.5) | 0.70 |
| PVE | 11 (52) | 5 (39) | 6 (75) | 0.18 |
| Surgery | 10 (48) | 6 (46) | 4 (50) | 1.0 |
| Suppressive therapy^b^ | 15 (79) | 8 (73) | 7 (88) | 0.60 |
| Treatment failure^c^ | 8 (38) | 7 (54) | 1 (12) | 0.10 |
| In-hospital mortality | 2 (10) | 2 (15) | - | 0.5 |
| Relapse | 6 (29) | 5 (38) | 1 (12) | 0.18^d^ |
| One-year mortality from index hospitalization^e^ | 3(19) | 2 (25) | 1 (12) | 0.61 |
| Data are presented as no (%). CCI, Charlson Comorbidity Index; IE, infective endocarditis; IQR, interquartile range; PVE, prosthetic valve endocarditis.  ^a^*p*-values for comparison between the two groups C. parapsilosis and C. non-parapsilosis.  ^b^(%) based on 19 episodes who were alive at discharge and thereby eligible for suppressive therapy.  ^c^Treatment failure defined as in-hospital mortality or relapse.  ^d^*p*-value based on 19 episodes alive at discharge.  ^e^(%) based on the 16 patients treated with amphotericin B-based backbone therapy at index hospitalization. | | | | |
